# Accumulation of N and P in the Legume *Lespedeza davurica* in Controlled Mixtures with the Grass *Bothriochloa ischaemum* under Varying Water and Fertilization Conditions

**DOI:** 10.3389/fpls.2018.00165

**Published:** 2018-02-13

**Authors:** Bingcheng Xu, Weizhou Xu, Zhi Wang, Zhifei Chen, Jairo A. Palta, Yinglong Chen

**Affiliations:** ^1^State Key Laboratory of Soil Erosion and Dryland Farming on the Loess Plateau, Northwest A&F University, Yangling, China; ^2^Institute of Soil and Water Conservation, Chinese Academy of Sciences and Ministry of Water Resources, Yangling, China; ^3^School of Life Sciences, Yulin University, Yulin, China; ^4^CSIRO Agriculture and Food, Wembley, WA, Australia; ^5^Institute of Agriculture, School of Agriculture and Environment, The University of Western Australia, Perth, WA, Australia

**Keywords:** intercropping, mixture proportion, soil water, N:P ratio, P fertilization

## Abstract

Water and fertilizers affect the nitrogen (N) and phosphorus (P) acquisition and allocation among organs in dominant species in natural vegetation on the semiarid Loess Plateau. This study aimed to clarify the N and P accumulation and N:P ratio at organ and plant level of a local legume species mixed with a grass species under varying water and fertilizer supplies, and thus to fully understand the requirements and balance of nutrient elements in response to growth conditions change of native species. The N and P concentration in the organ (leaf, stem, and root) and plant level of *Lespedeza davurica* (C_3_ legume), were examined when intercropped with *Bothriochloa ischaemum* (C_4_ grass). The two species were grown outdoors in pots under 80, 60, and 40% of soil water field capacity (FC), -NP, +N, +P, and +NP supply and the grass:legume mixture ratios of 2:10, 4:8, 6:6, 8:4, 10:2, and 12:0. The three set of treatments were under a randomized complete block design. Intercropping with *B. ischaemum* did not affect N concentrations in leaf, stem and root of *L. davurica*, but reduced P concentration in each organ under P fertilization. Only leaf N concentration in *L. davurica* showed decreasing trend as soil water content decreased under all fertilization and mixture proportion treatments. Stems had the lowest, while roots had the highest N and P concentration. As the mixture proportion of *L. davurica* decreased under P fertilization, P concentration in leaf and root also decreased. The N concentration in *L. davurica* at the whole plant level was 11.1–17.2%. P fertilization improved P concentration, while decreased N:P ratio in *L. davurica*. The N:P ratios were less than 14.0 under +P and +NP treatments. Our results implied that exogenous N and P fertilizer application may change the N:P stoichiometry and influence the balance between nutrients and organs of native dominant species in natural grassland, and P element should be paid more attention when considering rehabilitating degraded grassland via fertilization application in semiarid Loess Plateau region.

## Introduction

Plant growth and productivity in terrestrial ecosystems are limited by N and P (Aerts and Chapin, 2000; Güsewell, 2004; Vitousek et al., 2010; Dijkstra et al., 2016). Plant N demand is largely determined by photosynthetic N use efficiency, which varies among species because of leaf morphology or photosynthesis differences (Lambers and Poorter, 1992; Garnier et al., 1995). The variation in the requirements of P is related to internal phosphate recycling rates or related metabolic pathways differences among plants (Vance et al., 2003; Chen et al., 2013). N and P contents in plants are jointly affected by plant intrinsic properties, carbon synthesis characteristics and meteorological and pedologic factors (Li et al., 2007). In terrestrial ecosystems, N and P availability is important for community function and vegetation composition, and N:P ratios changing in plants may influence the competition balance among species in their communities (Güsewell et al., 2002; Agren, 2004; van der Hoek et al., 2004; Han et al., 2005; Fujita et al., 2010). The growth and productivity performance of species is affected by N and P fertilization, because plants need to maintain a certain N:P stoichiometric balance to function properly at the specific conditions (Fujita et al., 2010). Consequently, it is critical to determine the N and P requirements of each species in order to minimize interspecific competition and increase yield in their mixtures (Isaac et al., 2012; Neugschwandtner and Kaul, 2015, 2016).

High exploration of the root system for water and nutrient acquisition is required for improving N and P uptake efficiency when soil nutrients are scarce (Hinsinger et al., 2011a; Isaac et al., 2012). In limited nutrient environments, two or more species growing under intercropping or mixture plantations, may increase N and P acquisition and use efficiency, because such systems are supposed to enhance mutual positive interactions between plants (Zhang and Li, 2003; Li et al., 2007; Hinsinger et al., 2011a,b). In that respect, the cereal-legume intercropping system have advantages in growth, yield and resource use efficiency compared to respective sole cropping, because of their complementarity and facilitation in using light, water, and nutrition (Zhang and Li, 2003; Li et al., 2007; Hinsinger et al., 2011a; Betencourt et al., 2012; Xia et al., 2013; Tang et al., 2014; Neugschwandtner and Kaul, 2014, 2015). Although the improved acquisition of N under the cereal-legume intercropping is well known, only few recent studies indicated that similar effects may occur for P (Hinsinger et al., 2011a; Betencourt et al., 2012; Xia et al., 2013). In some dry environments, like the semi-arid Loess Plateau of China, the soil environment is hostile for plant growth as water deficits often occur and the soil erosion causes losses of N and P (Meng et al., 2008; Lü and Han, 2010; Liu et al., 2013; Niu et al., 2016). To understand the acquisition and allocation of limiting N and P in plants is useful to determine their adaptation strategy to the environment, and find appropriate measures to increase their productivity and improve their function in the region.

Native dominant species have great eco-adaptation to regional climatic and soil environment, and play an important role in ecological conservation and services in the region. Understanding the N and P relations in these species under different water and nutrient (i.e., N and P) availability is critical to clarify their co-existence mechanisms, and to choose appropriate measures to improve their productivity in natural grassland. In the natural grassland community of the semi-arid Loess Plateau of China, there are two co-dominant perennial species, the C_4_ grass *Bothriochloa ischaemum* and the C_3_ legume subshrub *Lespedeza davurica*. When they are grown in mixtures under different water and fertilizer levels, biomass production and water use efficiency are higher than when grown alone (Xu et al., 2013). The different mixtures and levels of water and fertilizer supply, particularly N and P combined, affect the N and P concentration in different organs of the grass *B. ischaemum* (Xu et al., 2016). Here, we report a study aimed to examine the N:P stoichiometry of the matched legume species *L. davurica*, from both the organ and whole plant level in response to soil water, mixture proportions and fertility changing. Our aims were to clarify the N and P accumulation and N:P ratio in *L. davurica* at organ and plant level, and examine the effects of soil water and N and P fertilizers on N:P stoichiometry in *L. davurica* intercropped with *B. ischaemum* in various mixture proportions.

## Materials and Methods

### Plant Material and Growth Conditions

The two co-dominant perennial species, *B. ischaemum* and *L. davurica* were used in this study. Their seeds were previously collected from a natural grassland community and tested for germination prior to this study. Seed collection was made at the Ansai Research Station, Ansai County, Shaanxi Province, China (36°51′30″N, 109°19′23″E). Plants were grown in cylindrical plastic pots 20 cm diameter, 30 cm depth, filled with 9.0 kg of the soil at a bulk density approx.1.2 g cm^-3^. The experiment was conducted at the nursery of the State Key Laboratory of Soil Erosion and Dryland Farming on the Loess Plateau, Yangling, Shaanxi Province, China (34°12′N, 108°7′E, altitude 530 m a.s.l.). The soil was a sandy-loam, collected from the upper 20 cm of a field site at the station. The soil gravimetric water content at field capacity (FC) and wilting point in the pot was 20.0 and 4.0%, respectively. The FC value (g H_2_O 100 g dry soil^-1^) was obtained using Wilcox method (Duan et al., 2010). Referring to world reference base for soil resources (FAO, 2006), the loess soil (called Huangmiantu locally) is classified as Calcaric Cambisol (Qiu et al., 2012). The organic matter of the soil was 2.7 g kg^-1^, total N content 0.17 g kg^-1^, total P content 0.63 g kg^-1^ and total K 19.7 g kg^-1^. The soil pH value was 8.5, and the content of total CaCO_3_ was 103 mg kg^-1^. The extractable N, P and K were 11.2, 6.55 and 94.8 mg kg^-1^, respectively. The soil was amended with or without N and/or P fertilizer as per fertility treatment. An open-end plastic pipe (2.0 cm diameter) was inserted inside each pot adjacent to the inner wall to supply water at 10 cm height from the bottom of each pot. The seeds were germinated on filter paper moistened with deionized water, in covered Petri-dishes, at room temperature for 7 days, before transplanting into the pots. Germinated seeds of each species were sown in 12 equally spaced dibbles in each pot on March 31, 2010. The scheme of plant arrangement within the pots was same as described in Xu et al. (2011). After emergence, seedlings were thinned to one plant per dibble (i.e., 12 plants per pot), and all pots were initially well watered during the seedling establishment. The soil surface of each pot was covered with 40 g of perlite to reduce soil evaporation. During the rainy days, the pots were covered by rainout shelter.

### Treatments

The treatments consisted of (1) six mixture proportions of *B. ischaemum: L. davurica*: 12:0, 10:2, 8:4, 6:6, 4:8, and 2:10. The mixture proportions were designed using a replacement series design (Connolly et al., 2001), in which 12 plants per pot of *L. davurica* (L) were replaced by *B. ischaemum* (B); (2) four fertilization treatments: non-NP (-NP), N supplied at 0.025 g kg^-1^ soil (+N), P supplied at 0.1 g kg^-1^ soil (+P) and combined N (0.025 g kg^-1^) and P (0.1 g kg^-1^) (+NP). N and P were applied as urea (CON_2_H_4_) and KH_2_PO_4_, respectively, and the amount was about 123 kg ha^-1^ (N) and 225 kg ha^-1^ (P), respectively; and (3) three soil water regimes: 16% (80% FC), 12% (60% FC), and 8% (40% FC). No other nutrients were applied as a base fertilization. The water treatments were imposed to match the natural grassland soil water content, because previous research has showed that the soil gravimetric water content in the natural grassland of the Loess Plateau region varies between 6 and 20% (Chen et al., 2008; Jiao et al., 2013). The mixture proportions, fertilization treatments and soil water regimes were arranged as a randomized complete block design. Each treatment had six replications (three in each harvest).

### Sampling

There were two harvests. The first sampling was made on June 21, 2010, 82 days after sowing (DAS), when plants of *B. ischaemum* were at 5th leaf stage and plants of *L. davurica* were branching. This sampling was made to calibrate the watering regimes only. Three pots for each mixture proportion (totally 18 pots) were harvested and the fresh biomass production of the two species was measured as reference. Then the three soil water content (SWC) regimes were induced by withholding watering and regularly weighing of the pots until they reached 80% FC, 60% FC, and 40% FC, respectively.

The second sampling was made on October 19, when the plants reached maturity at 202 DAS. This sampling was made to measure plant biomass by separating the shoots and roots. The roots were then washed to remove the soil. The leaves, stems and roots from each plant were separated and dried at 75°C for 48 h in an air-drying oven. In some treatments, *L. davurica* had seeds and ears, and which were combined into stem biomass and element analysis. The dry weight of each pot was combined as one biomass sample for each species.

### Soil Chemical Properties Measurement

The chemical properties of the soil used in the study were performed using standard soil test procedures from the Chinese Ecosystem Research Network (Editorial Committee, 1996) and the Soil Science Society of China (1999). Soil pH value was determined in a 1:2.5 soil/water slurry using a Delta 320 pH meter (Mettler-Toledo, Shanghai, China). Soil CaCO_3_ content was determined by using the Chittick apparatus (Dreimanis, 1962). Soil organic matter content was determined by wet digestion with a mixture of potassium dichromate and concentrated sulfuric acid. Soil total nitrogen content was measured via the semi-micro Kjeldahl method and a Kjeltec System 1026 Distilling Unit. Soil total phosphorus content was determined colorimetrically samples went through wet digestion with H_2_SO_4_+ HClO_4_. Total potassium was analyzed by the sodium hydroxide melting method with flame photometers. Soil available nitrogen content was determined with a micro-diffusion technique after samples went through alkaline hydrolysis. Soil available phosphorus was determined by the Olsen method (ISSCAS, 1978). Soil available potassium was measured in 1.0 mol L^-1^ NH_4_OAc extracts by flame photometry.

### Leaf, Stem, and Root N and P

N concentration in leaves, stems (including ears) and roots were determined using the Auto-Kjeldahl method (Kjektec System 2300 Distilling Unit, Foss, Sweden). Total P concentration in leaves, stems (including ears) and roots were analyzed using a molybdate/stannous chloride method after H_2_SO_4_–H_2_O_2_–HF digestion quantified by reference to a national standard material with a known P concentration (TU1901 UV spectrophotometer, China) (Qin et al., 2016). Plant tissues were grounded to a fine powder using a ring mill through 100-mesh. Total plant N and P concentration was determined by the summation of the N and P amount in each organ and then divided by the total biomass production. The N:P concentration ratio was calculated as the N concentration divided by the P concentration.

### Statistical Analysis

Differences in the mean N and P concentration and N:P concentration ratio for each organ and whole plant for each treatment (soil water regime, mixture proportion or fertilization, mixture vs. monoculture) were determined using the Tukey LSD analysis of SPSS Statistics17.0 (IBM, United States). Differences were considered significant for all statistical tests at *p* ≤ 0.05. The interactive effects of mixture proportion, fertilizer supply and soil water regime on N and P concentration and N:P ratio in each organ and whole plant was analyzed using a three-way ANOVA through GLM (general liner models) in SPSS.

## Results

### Temperature

The mean annual temperature at the State Key Laboratory of Soil Erosion and Dryland Farming on the Loess Plateau, Yangling, China was 13.0°C with a maximum mean temperature of 26.7°C in July and a minimum temperature of -1.5°C in January. Mean annual precipitation was 650 mm.

### Leaf, Stem, and Root N Concentration

**FIGURE 1:**
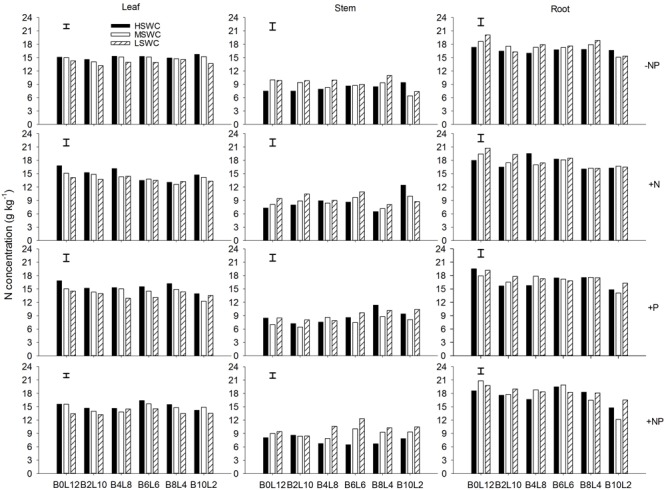
Leaf, stem, and root N concentration of *Lespedeza davurica* (L) mixed with *Bothriochloa ischaemum* (B) at various mixture proportions under three soil water regimes [HSWC: 80% FC (field capacity); MSWC: 60% FC; LSWC: 40% FC] and four fertilization treatments (–NP, +N, +P, +NP). B*i*L*j* (*i, j* = 0, 2, 4, 6, 8, 10, 12; *i*+*j* = 12) means the plant numbers of *B. ischaemum* to *L. davurica* in their mixtures. Error bars are LSD (*p* < 0.05).

Leaf N concentration in *L. davurica* decreased as soil water content decreased under all fertilization and mixture proportion treatments. Stem N concentration under 40% FC was higher than those under 80% FC and 60% FC soil water regimes. Root N concentration was higher in monocultures than in mixtures, especially at SWC of 40% FC. Under each treatment (i.e., soil water regime, fertilization, mixture proportion), stem had significantly lower while root had significantly higher (*p* ≤ 0.05) N concentration (**Figure 1**). Fertilization had no effects on stem N concentration, and the interactions of SWC × fertilization on leaf and root N concentration, and the interaction of SWC × mixture proportion on leaf N concentration of *L. davurica* were not significant (**Table 1**).

**Table 1:** *P*-values of three-way ANOVA to evaluate the effect of soil water content (SWC), fertilization treatment (FT), mixture proportion (MP) and their interactions on N and P concentration in leaves, stems, roots and whole plants of *Lespedeza davurica* intercropped with *Bothriochloa ischaemum*.

Source	df	Leaf			Stem			Root			Whole plant		
		LNC (%)	LPC (%)	N:P	SNC (%)	SPC (%)	N:P	RNC (%)	RPC (%)	N:P	WNC (%)	WPC (%)	N:P
Soil water content (SWC)	2	**<0.001**^∗^	**<0.001**	**0.001**	**<0.001**	**<0.001**	0.127	**<0.001**	**<0.001**	**<0.001**	**<0.001**	**<0.001**	**<0.001**
Fertilization treatment (FT)	3	**0.010**	**<0.001**	**<0.001**	0.116	**<0.001**	**<0.001**	**<0.001**	**<0.001**	**<0.001**	**<0.001**	**<0.001**	**<0.001**
Mixture proportion (MP)	5	**<0.001**	**<0.001**	**<0.001**	**<0.001**	**<0.001**	**<0.001**	**<0.001**	**<0.001**	**<0.001**	**<0.001**	**<0.001**	**<0.001**
SWC × FT	6	0.168	0.084	0.065	**<0.001**	0.419	0.167	0.898	**0.002**	**<0.001**	**0.003**	**0.032**	**<0.001**
SWC × MP	10	0.588	0.272	0.414	**<0.001**	**<0.001**	**0.010**	**0.001**	**<0.001**	**0.001**	**<0.001**	**<0.001**	**0.006**
FT × MP	15	**<0.001**	**<0.001**	**<0.001**	**<0.001**	**<0.001**	**<0.001**	**<0.001**	**<0.001**	**<0.001**	**<0.001**	**<0.001**	**<0.001**
SWC × FT × MP	30	**0.004**	0.077	0.084	**<0.001**	**<0.001**	**0.002**	**<0.001**	**<0.001**	**<0.001**	**<0.001**	**<0.001**	**<0.001**

*^∗^Probabilities considered statistically significant are indicated in bold typeface (p ≤ 0.05).*

*LNC, leaf N concentration; LPC, leaf P concentration; SNC, stem N concentration; SPC, stem P concentration; RNC, root N concentration; RPC, root P concentration; WNC, whole plant N concentration; WPC, whole plant P concentration; N:P, N:P ratio.*

### Leaf, Stem, and Root P Concentration

**FIGURE 2:**
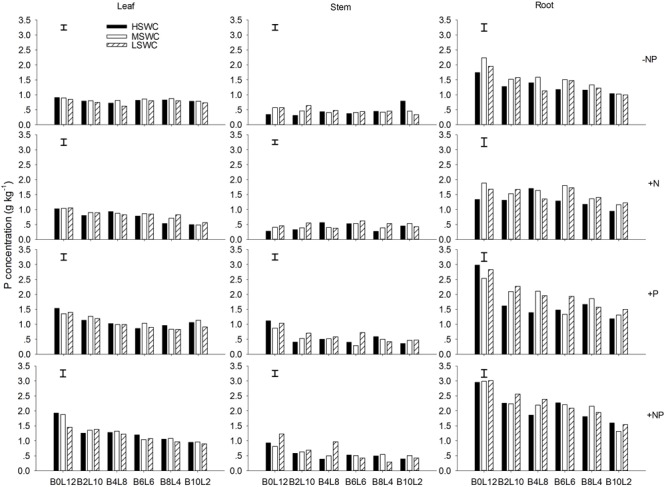
Leaf, stem, and root P concentration of *L. davurica* (L) mixed with *B. ischaemum* (B) at various mixture proportions under three soil water regimes [HSWC: 80% FC (field capacity); MSWC: 60% FC; LSWC: 40% FC] and four fertilization treatments (–NP, +N, +P, +NP). B*i*L*j* (*i, j* = 0, 2, 4, 6, 8, 10, 12; *i*+*j* = 12) means the plant numbers of *B. ischaemum* to *L. davurica* in their mixtures. Error bars are LSD (*p* < 0.05).

The leaf P concentration of *L. davurica* were higher under monoculture than under mixtures (*p* < 0.05), and showed a decreasing trend as its ratio in the mixture decreased (**Figure 2**). P fertilizer (+P and +NP treatments) significantly improved leaf P concentration across mixture proportions and soil water regimes (**Figure 2**). For each SWC, fertilization or mixture proportion treatment, the significantly highest P concentration was found in the roots and the lowest in the stems. The interaction of SWC and fertilization did not have effects on P concentration in both leaf and stem. The interactions from SWC and mixture proportion, and SWC × mixture proportion × fertilization interaction did not affect leaf P concentration. P concentration in root was significantly affected by SWC, mixture proportion or fertilization, and their combined interactions (**Table 1**).

### Leaf, Stem, and Root N:P Ratio

**FIGURE 3:**
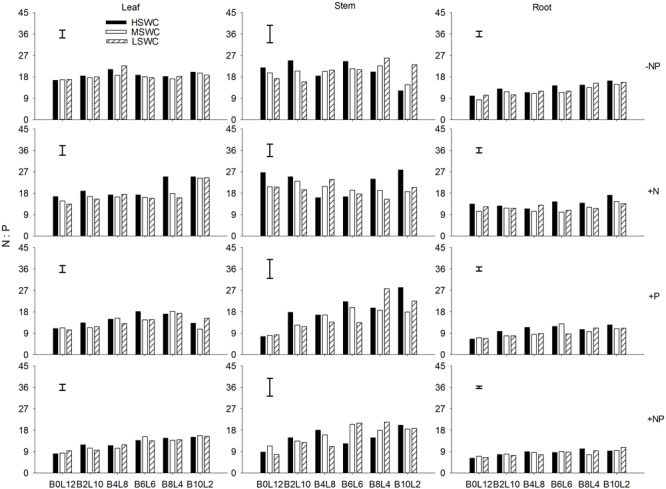
Leaf, stem, and root N:P ratios of *L. davurica* (L) mixed with *B. ischaemum* (B) at various mixture proportions under three soil water regimes [HSWC: 80% FC (field capacity); MSWC: 60% FC; LSWC: 40% FC] and four fertilization treatments (–NP, +N, +P, +NP). B*i*L*j* (*i, j* = 0, 2, 4, 6, 8, 10, 12; *i*+*j* = 12) means the plant numbers of *B. ischaemum* to *L. davurica* in their mixtures. Error bars are LSD (*p* < 0.05).

P fertilization decreased the N:P ratio in each organ of *L. davurica* under all water regimes in monocultures or mixtures (**Figure 3**). When N was applied (+N and +NP treatments), N:P ratio increased gradually as the mixture proportion of *L. davurica* decreased. The N:P ratio in the stem was higher while in the root it was lower under all soil water regime, mixture proportion and fertilization treatments. Soil water content had no effect on stem N:P ratio, and its interaction with fertilization did not have effects on N:P ratio in leaf and stem (**Table 1**). No significant effects of the interactions from SWC and mixture proportion, and interaction of SWC, mixture proportion and fertilization on leaf N:P ratio were detected.

### Whole Plant N and P Concentration

**FIGURE 4:**
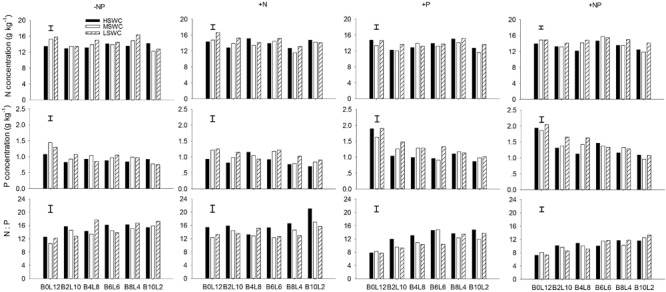
Whole plant N concentration, P concentration and N:P ratio of *L. davurica* (L) mixed with *B. ischaemum* (B) at various mixture proportions under three soil water regimes [HSWC: 80% FC (field capacity); MSWC: 60% FC; LSWC: 40% FC] and four fertilization treatments (–NP, +N, +P, +NP). B*i*L*j* (*i, j* = 0, 2, 4, 6, 8, 10, 12; *i*+*j* = 12) means the plant numbers of *B. ischaemum* to *L. davurica* in their mixtures. Error bars are LSD (*p* < 0.05).

At the whole plant level, the N concentration in *L. davurica* was 11.1–17.2%. The P concentration had a tendency to decrease when the proportion of *L. davurica* in the mixtures decreased, especially under P fertilizer supply (+P or +NP) treatments (**Figure 4**). P fertilizer also significantly increased P concentration in *L. davurica* for each mixture proportion and soil water regime. Only in a few cases, the values of N:P ratio were bigger than 16.0, and all appeared in -NP and +N fertilization treatments. Under P fertilizer supply (+P or +NP) treatments, the N:P ratio concentration had a tendency to increase when the proportion of *L. davurica* in the mixtures decreased, and as the N:P ratio concentration were smaller than 16.0, and lower than the values under -NP and +N treatments for each mixture proportion and soil water regime (**Figure 4**). SWC, fertilization treatment, mixture proportion, and their interaction significantly affected N and P concentration and N:P ratio in *L. davurica* (**Table 1**).

### N and P Allocation in Single Plant

**FIGURE 5:**
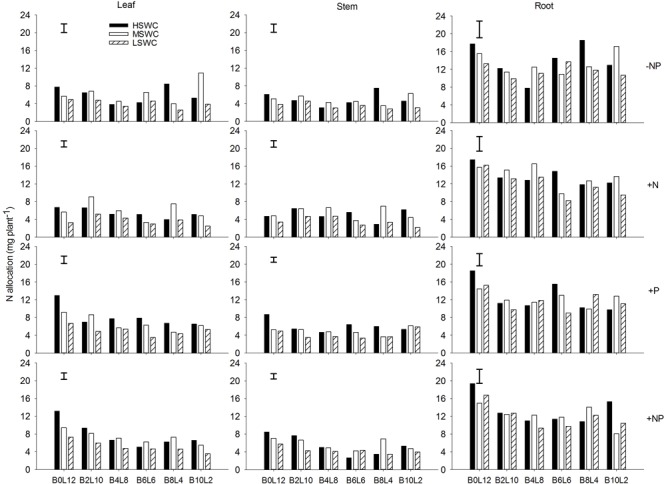
N allocation in leaf, stem and root of *L. davurica* (L) mixed with *B. ischaemum* (B) at various mixture proportions under three soil water regimes [HSWC: 80% FC (field capacity); MSWC: 60% FC; LSWC: 40% FC] and four fertilization treatments (–NP, +N, +P, +NP). B*i*L*j* (*i, j* = 0, 2, 4, 6, 8, 10, 12; *i*+*j* = 12) means the plant numbers of *B. ischaemum* to *L. davurica* in their mixtures. Error bars are LSD (*p* < 0.05).

**FIGURE 6:**
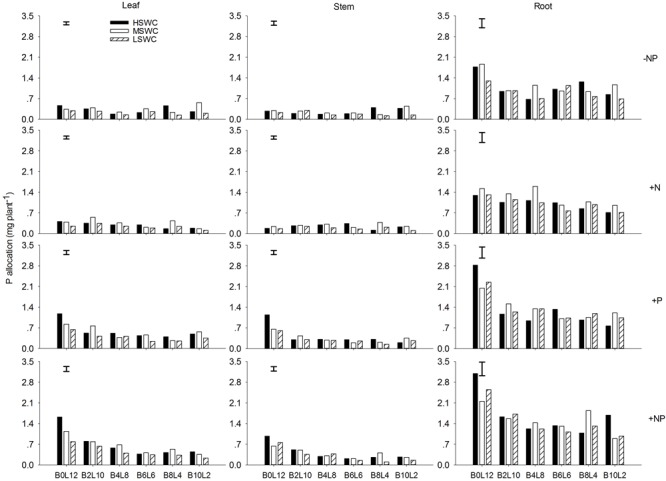
P allocation in leaf, stem and root of *L. davurica* (L) mixed with *B. ischaemum* (B) at various mixture proportions under three soil water regimes [HSWC: 80% FC (field capacity); MSWC: 60% FC; LSWC: 40% FC] and four fertilization treatments (–NP, +N, +P, +NP). B*i*L*j* (*i, j* = 0, 2, 4, 6, 8, 10, 12; *i*+*j* = 12) means the plant numbers of *B. ischaemum* to *L. davurica* in their mixtures. Error bars are LSD (*p* < 0.05).

In the single plant level, N and P acquired by *L. davurica* under all treatments were mainly allocated to roots (**Figures 5, 6**). The average amount across mixture proportions and water regimes of N allocation in leaf, stem and root were 3.71–8.15, 3.49–6.08, and 11.68–13.96 mg plant^-1^, respectively. The mean amount of P in leaf, stem and root of single plant was 0.22–0.71, 0.18–0.43, and 0.93–1.67 mg plant^-1^ across all mixture proportions and water regimes, respectively. Under P fertilizer (+P and +NP treatments), P amount in leaf, stem and root was significantly improved. P fertilization significantly increased the N amount in leaf and stem (**Figures 5, 6**).

### Whole Plant Biomass Production and Partitioning

Individual plant biomass production of *B. ischaemum* decreased as its proportion increased in the mixtures, and the monoculture had the smallest values (Supplementary Figure S1). Soil water content significantly affected biomass production of *B. ischaemum*, regardless of mixing proportions and fertilizer treatments. Across mixture proportions including monoculture, the averaged biomass production under 40% FC was significantly lower (*p* < 0.05) than under 80% FC and 60% FC, and there was no significant differences between the latter two (Supplementary Figure S2). Single plant biomass production of *L. davurica* averagely increased about 10% under fertilizer applications (+N, +P, or +NP treatments). The averaged root biomass in the whole plant accounted for more than 38% across mixture proportions and fertilizations (Supplementary Figure S3). Leaf biomass accounted for 20.8–27.1% of the whole plant of *L. davurica* in the experiment, and showed obvious decreasing trend as its proportion decreased in the mixture under +NP treatment.

## Discussion

Legume and grass growing together is a common phenomenon in natural community. However, less attention has been given to nutrient behavior in different organs in their mixtures. Together with the sister paper, the studies provides the evidences of the N:P stoichiometric changes with mixture proportions in grass-forage mixture systems of native species in semiarid Loess Plateau (Xu et al., 2016). N and P concentration in different organs of the legume *L. davurica* varied with the SWC and fertilization when mixed with *B. ischaemum*, but their changing patterns were different. Leaf and stem N concentration of *L. davurica* did not vary significantly in response to mixture proportions, which may be associated to the low N requirements of this leguminous specie or it can maintain relatively stable N concentration in both organs under different conditions (Fujita et al., 2010; Sardans et al., 2012). In the grass-legume mixtures, it is likely that the grass absorbed N that was released by the legume root and nodule root system, resulting in a more efficient use of nutrients (Hauggaard-Nielsen and Jensen, 2005; Chen et al., 2013; Neugschwandtner and Kaul, 2014, 2015). Here N concentration in the roots of *L. davurica* under sole cropping was higher than under intercropping, indicating that mixture plantation decreased N enrichment in roots of *L. davurica* (**Figure 1**). It also may be caused by strong N acquisition of the neighboring shallow-root grass species *B. ischaemum*, whose fibrous root system intermingled with the legume roots and acquired the legume-derived N (Xu et al., 2016; Dhamala et al., 2017).

P concentrations in different organs of *L. davurica* under intercropping decreased compared with its monoculture, especially under +P or +NP treatments. There were two likely processes that may influence P concentration in *L. davurica*, as its proportion decreased in the mixtures: the first was that P fertilization tended to increase P availability, and the second was that limited P resources from fertilizer and soil was equally used by each single plant of *L. davurica*. Therefore, the increased numbers in the mixtures would decrease the P reallocation, limiting plant P uptake and P concentration in organs of *L. davurica* (Mi et al., 2015). Similar results were reported for the soybean-citrus intercropping, where P uptake and distribution amongst the organs of soybean decreased compared with their respective monoculture (Zhou et al., 2006). This contrasts with what was expected that P acquisition would be improved in both legumes and cereals under their mixtures because soil rhizosphere pH decreased, and P activities in the soils and roots (e.g., rhizosphere soil acid phosphatase activities) and rhizosphere soil P concentration increased (Inal et al., 2007; Betencourt et al., 2012). Unfortunately, we did not measure soil rhizosphere pH and phosphatase activity in the soils and roots in this study. P concentration in different organs or whole plant level of *L. davurica* decreased as its numbers decreased in the mixtures, implying that intraspecific competition intensification may increase its P uptake.

The higher N or P concentrations in the roots than in other organs and the higher N or P concentrations in the leaves than in the stems of *L. davurica*, could be explained by competition between the plant organs for N and P demand for growth, and the advantage that an organ may have for being close to the nutrient source. This is because nutrients are allocated to distant plant organs, after the demand of the adjacent organs to the nutrient source are met (Yang et al., 2014). However, this contradicts the findings from shrubs, where nutrient concentration in the roots increased in parallel with their neighbors, the stem and the leaves (Yang et al., 2014). Nutrient allocation is also driven by the plant and organ demand for N-rich proteins and P-rich RNAs (Vrede et al., 2004). Additional N and P allocation to the leaves are important to maintain leaf photosynthesis and photosynthate remobilization (Li et al., 2010). In dry environments (or dry years), a high N concentration in the leaves may provide adaptation to drought and competition by exploiting greater light availability while also increasing water use efficiency (Wright et al., 2003; Li et al., 2010; Yang et al., 2014).

Leaf N:P concentration ratio was higher than 16.0 under -NP and +N treatments, indicating a P limitation (Koerselman and Meuleman, 1996; Tessier and Raynal, 2003). Across most treatments (e.g., soil water regime, mixture proportion and fertilization), N:P ratio in leaf and root was significantly lower than 14.0, indicating a N limitation (Craine et al., 2008; Li et al., 2010). At whole plant level, the N:P ratio values were between 14.0 and 16.0 under -NP and +N treatments, while significantly smaller than 14.0 under +P and +NP treatments. These suggest that the species may have low P requirements, and mixture and fertilization changed the P accumulation in *L. davurica* (Zhou et al., 2006; Hinsinger et al., 2011a). Normally, N:P ratio is higher in structural organs than in metabolically active organs, as indicated here root had significantly lower N:P ratio (Xiao et al., 2004; Kerkhoff et al., 2006; Xu et al., 2013).

Our previous paper showed that the aggressivity values of *B. ischaemum* were bigger than 0 in their mixtures, which meant that *B. ischaemum* was the dominant while *L. davurica* the dominated species in their mixtures. In the study, the pot depth was about 30 cm, and the main soil depth for their root distribution was in 0–20 cm soil depth, which would strengthen the interaction between their root systems (Xu et al., 2015). This would explain why P facilitation may occur rather than competition between the two-species under intercropping. It is likely, that *L. davurica* was the preferred user of this pool when intercropped with *B. ischaemum* in comparison with respective monoculture (Li et al., 2008; Xu et al., 2013). Another reason could be that strong competition between them for accessing the same soil horizons because both are native species whose roots mainly distribute in the top soil layer and the small pots may restrict root distribution and proliferation (Li et al., 2006; Zhou et al., 2006; Hinsinger et al., 2011a).

Water is the main factor limiting plant growth and production in arid and semiarid region on the Loess Plateau of China. Nutrient uptake and concentrations in plants would be reduced by drought due to decreased transpiration flow and root growth, and lower soil nutrient mobility (Neugschwandtner and Kaul, 2015, 2016). In the specific water condition, rational fertilization is favorable for plant growth and production (Shan and Chen, 1993; Xu et al., 2016). Here, we found that single plant biomass production of *L. davurica* increased about 10% under N and/or P fertilization treatments, while the effects were mixture ratio-specific (Supplementary Figure S3). The N and P concentration in organ and whole plant level generally increased as N and/or P fertilizer were applied, while they were not exactly the same for all treatments. Along or with N, P fertilization could not only increased biomass production, but also nutrient concentration in each organ of *L. davurica* across mixture proportions and soil water regimes. For example, compared with 40%FC soil regime, single plant biomass production of *L. davurica* under 80%FC increased 35.8% at 10:2 of *B. ischaemum* to *L. davurica* after +NP treatment, while N concentration in leaf and whole plant level decreased 10 and 12%, respectively (**Figures 1, 4, 5**). In the same mixture ratio, compared with 80%FC, the single plant biomass production of *L. davurica* under 40% FC soil regime decreased about 27% after N fertilization (i.e., +N treatment), but N concentration in whole plant level increased about 10.7% (**Figures 1, 5**). All these suggested that P fertilization played an important function in improving biomass production and increasing nutrient concentrations in different organs of the legume, and also implied that the improvement of biomass production after fertilization may possess “dilution effect” on nutrient concentration in plant, besides “enrichment effect” (Craine et al., 2008; Neugschwandtner and Kaul, 2015; Li et al., 2016).

## Conclusion

On the semiarid Loess Plateau of China, wild and native plant species experience the harsh environment with variable soil water and infertile soil nutrient conditions. Artificial fertilization was normally applied in order to increase their growth, and thus to improve vegetation coverage, accelerate successional process and mitigate desertification in the region. Results showed that P applied alone or combined with N, affected not only the biomass production, but also the N and P accumulation in each component of *L. davurica* in the mixtures, suggesting that P element should not be neglected when considering rehabilitating degraded grassland through fertilizer application. N and/or P fertilizers significantly affected N and P concentrations at both organ and whole plant levels, and the effects were closely related with mixture proportions, implying that exogenous fertilization may change the N:P stoichiometry of native dominant species in natural grasslands, and such effects may depend on their relative abundance in the community. Our results also provided clearly evidence of inconsistent changes of N:P stoichiometry in the organ and whole plant level under same treatment, suggesting that the organ differences should be considered when studying nutrient absorption and cycling between or among plant species. Because *B. ischaemum* and *L. davurica* are perennial species, and here we only reported the nutrient behaviors during the establishment period in their controlled mixtures, it is worthy and necessary to test their nutrient requirements as growth years extended, especially in the field natural conditions. Further research also needs to be taken on the nutrient cycling and balance at the specific mixture proportion in response to water and fertilization treatments to clarify the detailed mechanisms behind.

## Author Contributions

BX and YC conceived and designed the experiments. WX, ZW, and ZC performed the experiments. WX, ZW, ZC, and JP analyzed the data. BX and JP contributed reagents/materials/analysis tools. BX and YC wrote the paper.

## Conflict of Interest Statement

The authors declare that the research was conducted in the absence of any commercial or financial relationships that could be construed as a potential conflict of interest.
