# Supplementary material for: Accumulation of N and P in the Legume Lespedeza davurica in Controlled Mixtures with the Grass Bothriochloa ischaemum under Varying Water and Fertilization Conditions
Source: Front Plant Sci. 2018 Feb 13;9:165. doi: 10.3389/fpls.2018.00165 (PMC5816928; doi:10.3389/fpls.2018.00165)
Supplement: Supplementary file 1 [file Presentation_1.pdf]

Supplementary materials

Fig. S1 Biomass production (g $plant^{-1}$) of *Bothriochloa ischaemum* (B) mixed with *Lespedeza davurica* (L) at various mixture proportions under three soil water regimes [HSWC: 80 % FC (field capacity); MSWC: 60 % FC; LSWC: 40 % FC] and four fertilization treatments (−NP, +N, +P, +NP). The vertical bars indicates the LSD value ($p \leqslant 0.05$) for differences between soil water content (SWC) for each mixture proportion. B*i*L*j* (*i*, *j*=0, 2, 4, 6, 8, 10, 12; *i*+*j*=12) means the plant numbers of *B. ischaemum* to *L. davurica* in their mixtures.

Fig. S2 Biomass production (g $plant^{-1}$) of *Lespedeza davurica* (L) mixed with *Bothriochloa ischaemum* (B) at various mixture proportions under three soil water regimes [HSWC: 80 % FC (field capacity); MSWC: 60 % FC; LSWC: 40 % FC] and four fertilization treatments (−NP, +N, +P, +NP). The vertical bars indicates the LSD value ($p \leqslant 0.05$) for differences between soil water content (SWC) for each mixture proportion. B*i*L*j* (*i*, *j*=0, 2, 4, 6, 8, 10, 12; *i*+*j*=12) means the plant numbers of *B. ischaemum* to *L. davurica* in their mixtures.

Fig. S3. Biomass partition of single plant of *Lespedeza davurica* (L) mixed with *Bothriochloa ischaemum* (B) at various mixture proportions under three soil water regimes [HSWC: 80 % FC (field capacity); MSWC: 60 % FC; LSWC: 40 % FC] and four fertilization treatments (−NP, +N, +P, +NP). B*i*L*j* (*i*, *j*=0, 2, 4, 6, 8, 10, 12; *i*+*j*=12) means the plant numbers of *B. ischaemum* to *L. davurica* in their mixtures. Error bars are LSD ($p<0.05$).

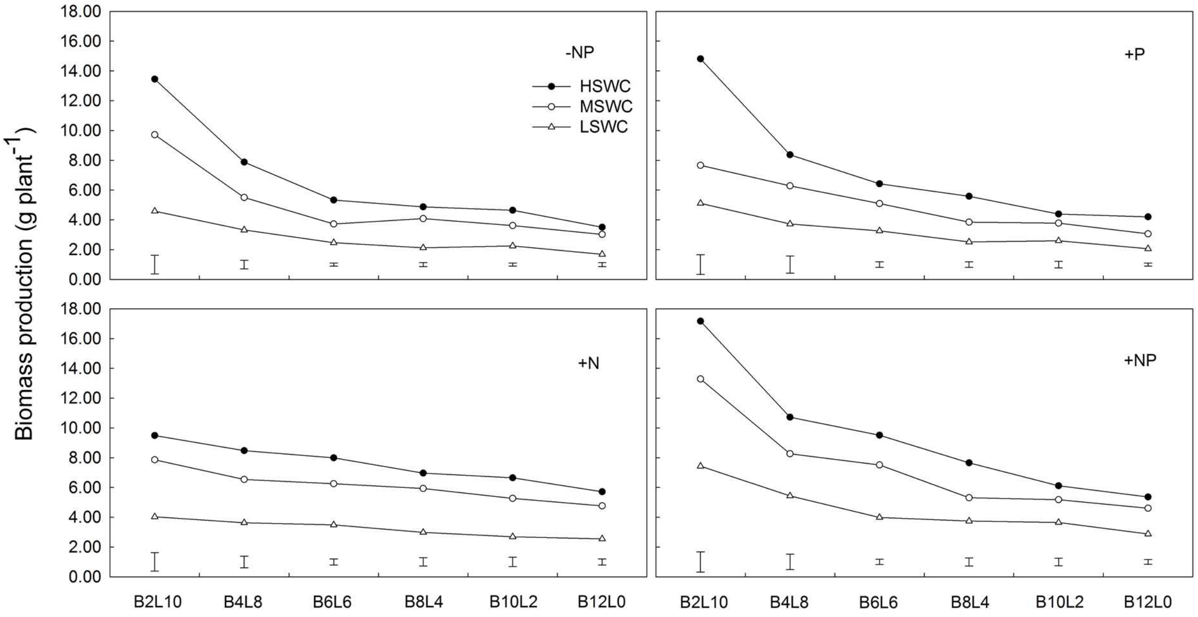

Biomass production (g plant-1)
-NP
HSWC
MSWC
LSWC
+P
+N
+NP
B2L10
B4L8
B6L6
B8L4
B10L2
B12L0

Fig. S1

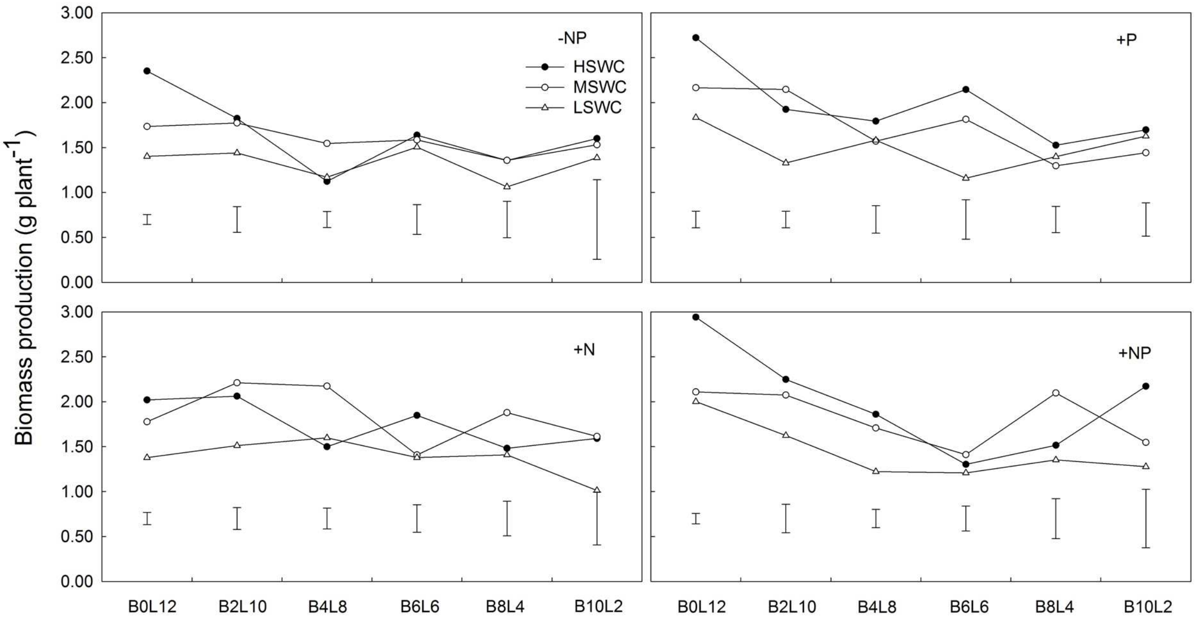

-NP
HSWC
MSWC
LSWC
+P
+N
+NP
Biomass production (g plant-1)
3.00
2.50
2.00
1.50
1.00
0.50
0.00
B0L12
B2L10
B4L8
B6L6
B8L4
B10L2

Fig. S2

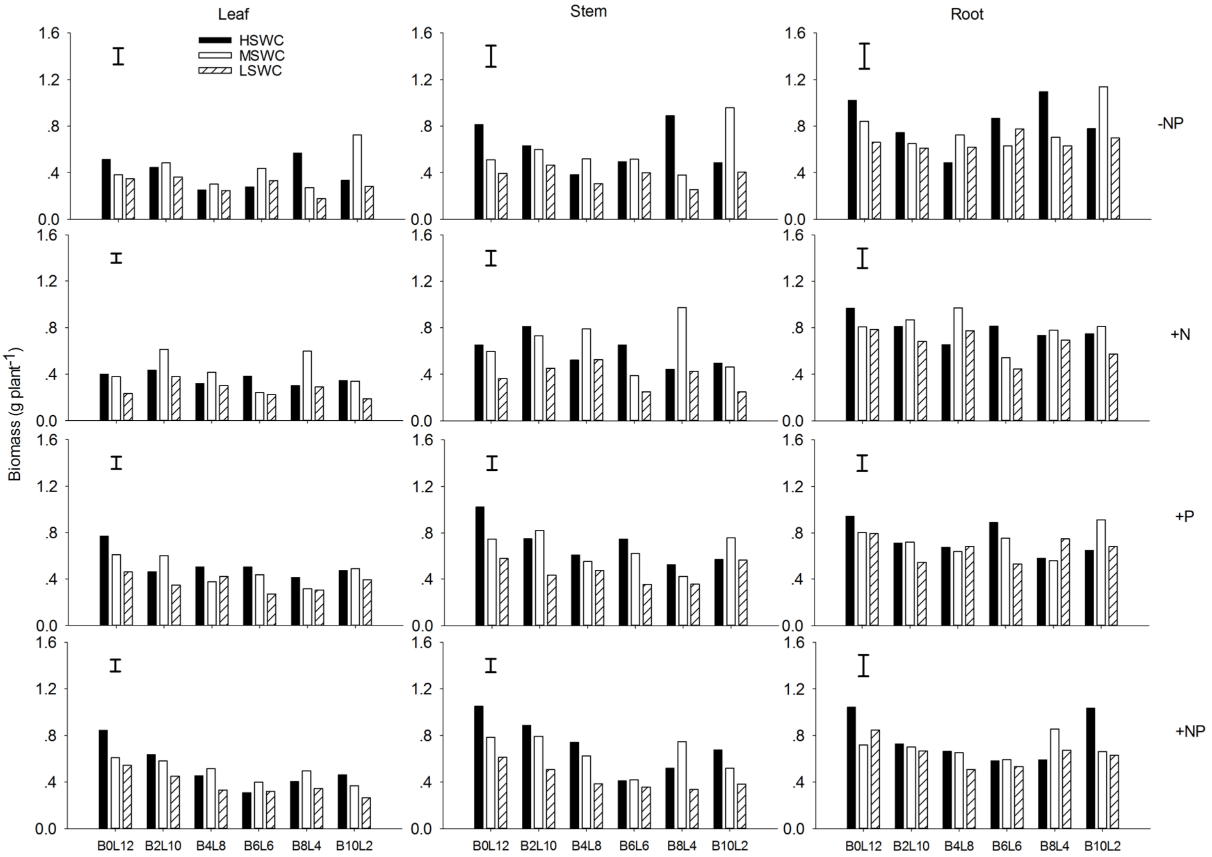
Leaf
Stem
Root
HSWC
MSWC
LSWC
-NP
+N
+P
+NP
Biomass (g plant-1)
B0L12
B2L10
B4L8
B6L6
B8L4
B10L2

Fig. S3
